# Vaso-Occlusive Crises in Sickle Cell Trait Patients With Blood Loss Anemia: A Report of Two Cases

**DOI:** 10.7759/cureus.56589

**Published:** 2024-03-20

**Authors:** Michael Sabina, Zein Barakat, Jennifer M Jost, Rachel Tatro, Wan Sai

**Affiliations:** 1 Internal Medicine, Lakeland Regional Health Medical Center, Lakeland, USA; 2 Research, Kiran C. Patel College of Osteopathic Medicine, Nova Southeastern University, Clearwater, USA

**Keywords:** uterine fibroid, packed red blood cell transfusion, menorrhagia, acute blood loss anemia, sickle cell disease: scd, vaso-occlusive crisis, sickle cell trait

## Abstract

This report of two cases confronts the longstanding perception of Sickle Cell Trait (SCT) as a clinically benign condition, highlighting its complex and severe clinical manifestations, particularly in the context of blood loss anemia and vaso-occlusive crises (VOCs). The hallmark of sickle cell disease is the severe pain caused by acute vaso-occlusion of the microvasculature that leads to bone marrow infarction. We report two cases of patients with SCT and severe anemia in the setting of blood loss secondary to uterine fibroids subsequently causing VOCs with likely bone sequestration. The occurrence of VOCs in SCT, while infrequent, can be serious and demands a high index of suspicion, particularly when patients appear in significant distress and cardiac or vascular etiologies are ruled out as a source. Reversal of anemia in this case provided quick resolution to symptoms, and we recommend other clinicians not disregard a differential of VOC in SCT carriers, and urge to treat patients as they would if they had sickle cell disease. This report challenges the conventional view of SCT as a condition of clinical benignity, calling for a recalibration in the clinical understanding, management strategies, and focus on this genetic trait under similar circumstances.

## Introduction

In the United States, 2.5 million to 3 million persons live with sickle cell trait (SCT) [1]. Sickle cell trait is the term used to describe individuals with a heterozygous glutamic acid-to-valine substitution in the β-globin gene on chromosome 11 (HbAS) [2]. Individuals possessing one copy of the βS allele (HbAS) demonstrate the sickle cell trait without developing sickle cell disease (HbSS). Conversely, those with two copies of the βS allele, being homozygous, suffer from sickle cell anemia (SCA), which is the predominant form of sickle cell disease (HbSS) [3]. SCA is a lifelong disease characterized by chronic hemolytic anemia and unpredictable episodes of vaso-occlusive crisis by way of erythrocytes polymerizing and assuming a sickled form under low oxygen tension states. The usual treatment is with hydroxycarbamide and blood transfusions which can reduce the severity of the disease [3]. This is generally thought to not be a complication in SCT (HbAS) carriers, who have a mixture of normal HbA and HbS molecules [4].

The overarching narrative of sickle cell trait’s (SCT's) clinical benignity is being continuously challenged. A case-control study revealed that African Americans with SCT have roughly double the risk of experiencing venous thromboembolism compared to those with the normal genotype [5]. Similarly, a 2015 prospective study indicated that the presence of SCT in African Americans is associated with a doubled likelihood of pulmonary embolism [6]. A systematic review of cases of sickle cell trait carrier patients with complications showed it is an independent risk factor for health outcomes, such as pulmonary embolism, kidney disease, and exertional rhabdomyolysis [1]. In 2010, following the death of a 19-year-old freshman due to exertional rhabdomyolysis linked to an undiagnosed case of SCT, the National Collegiate Athletic Association (NCAA) mandated SCT testing for Division I athletes [7,8]. This requirement was expanded to include Division II and III athletes in 2012, as a reaction to additional fatalities associated with SCT [9]. High-altitude conditions, known to cause hypobaric hypoxia, increase the risk for individuals with SCT to develop acute mountain sickness (AMS), high-altitude cerebral edema (HACE), and high-altitude pulmonary edema (HAPE) [10]. In response to SCT-related fatalities in the military, the 2021 Summit on Exercise Collapse Associated with Sickle Cell Trait was established. This summit aimed to offer education, update practice guidelines, and highlight areas needing attention for this vulnerable group [11].

Under hypoxic states, SCT red blood cells (RBCs) demonstrate pathophysiological changes that may promote hemoglobin polymerization and consequent splenic infarction and impairment of microvascular flow. Anemia intensifies the demand for oxygen delivery to tissues, potentially accelerating the occurrence of vaso-occlusive crises (VOCs). Positioning blood loss anemia within this narrative enhances the clinical complexity observed in SCT carriers.

## Case presentation

Case #1

A 38-year-old female with a past medical history of sickle cell trait (SCT) diagnosed at birth in the setting of paternal sickle cell disease, recurrent deep vein thrombosis (DVT), uterine fibroids, and a longstanding history of abnormal uterine bleeding that began in her 20s, presented with complaints of extreme lower extremity pain with swelling, and generalized weakness. She had a previous medical encounter where she underwent a dilation and curettage (D&C) procedure to address uterine bleeding three weeks prior to her current admission with an outpatient gynecologist. Despite this intervention, her symptoms persisted. Physical examination revealed pallor. Vital signs were stable with no hemodynamic compromise or hypoxia. Notably, her hemoglobin level was alarmingly low at 6.7 g/dL with a mean corpuscular volume (MCV) of 82.3, consistent with normocytic anemia.

**Figure 1:**
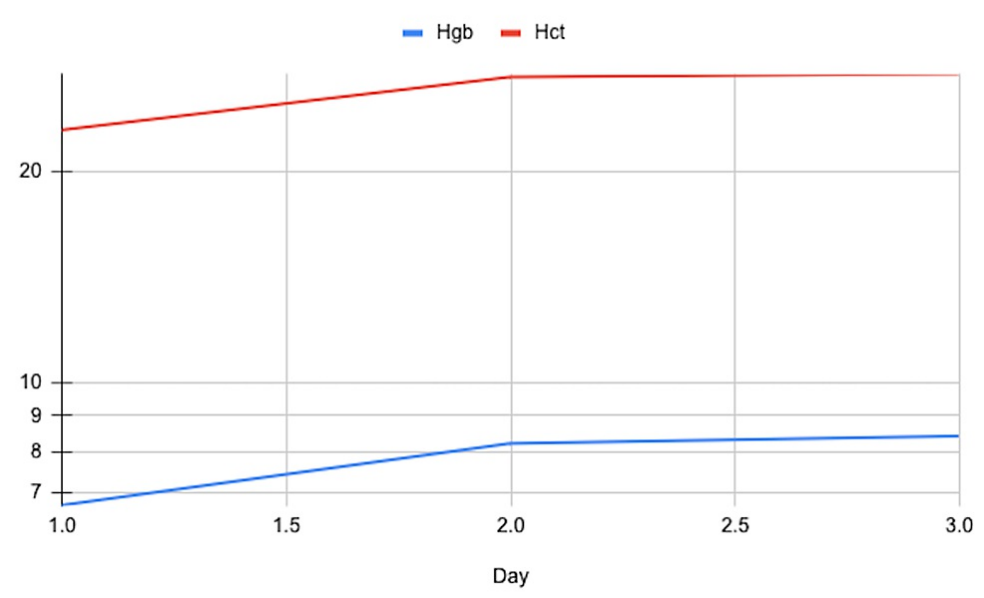
Case #1 Hemoglobin (Hgb) and Hematocrit (Hct) Trend

The patient was treated with two units of packed red blood cells (PRBCs) which led to a notable improvement in her symptoms, including pain and swelling reduction and normalization of her hemoglobin level (Figure 1). The obstetrics/gynecology (OBGYN) team was consulted to explore the possibility of uterine fibroids contributing to her recurrent anemia. A transvaginal ultrasound was conducted, revealing the presence of a fibroid (2 cm in diameter) within the body of the uterus. Simultaneously, the patient's past history of recurrent DVT and acute presentation raised concerns about the possibility of a thrombotic event. Doppler ultrasound studies of both the veins and arteries of the lower extremities were negative for deep vein thrombosis (DVT) and peripheral arterial disease, respectively.

Ultimately, we decided to proceed with prophylactic measures to treat a presumed vaso-occlusive crisis in the setting of blood loss anemia-induced VOC. An additional unit of PRBCs was administered as a prophylactic measure, and the patient was closely monitored for any further complications. Following this intervention and the stabilization of her hemoglobin levels, the patient experienced significant relief from her initial lower extremity pain and swelling. With her condition improving, she was prepared for discharge and provided with recommendations for follow-up care, including a scheduled evaluation with a hematologist to discuss ongoing management strategies for her SCT and recurrent anemia and an evaluation with her gynecologist for management of uterine fibroids.

Case #2

A 33-year-old female with a medical history of sickle cell trait diagnosed at an early age due to paternal history of sickle cell disease and iron deficiency anemia presented with worsening shortness of breath and fatigue. She has never had a vaso-occlusive crisis in the past. The patient had been having 3 days of heavy menstrual bleeding, with multiple clots, necessitating the use of diapers. She has tried iron supplements in the past for her deficiency but could not tolerate it due to severe constipation. Upon initial assessment, she appeared in mild distress but was hemodynamically stable, and oxygen saturation was 98%. Her labs indicated a critically low hemoglobin level of 6.8 g/dL, MCV 63.8 fL, and low iron levels (serum iron of 25 μg/dL and ferritin of 8.1 ng/mL), suggesting iron deficiency anemia. Her baseline hemoglobin levels could not be found as she had never been hospitalized and we did not have access to outside records.

**Figure 2:**
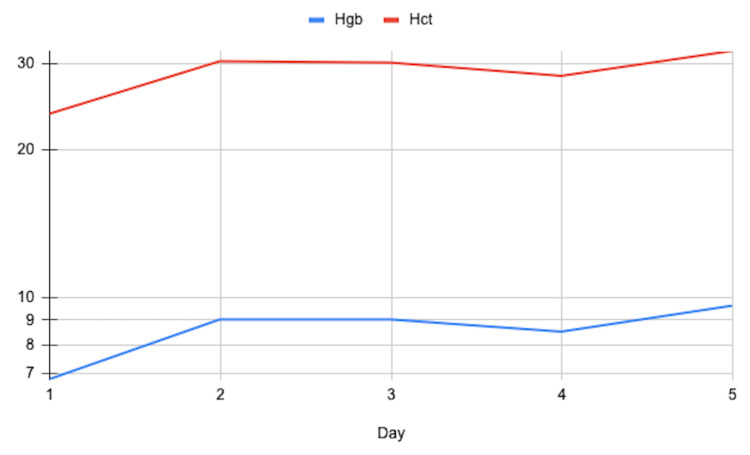
Case #2 Hemoglobin (Hgb) and Hematocrit (Hct) Trend

The initial plan included hemoglobin and hematocrit monitoring, blood transfusion if hemoglobin fell below 7 g/dL, an obstetrics/gynecology (OBGYN) consult, a hematology-oncology (HEME-ONC) consult, and a pelvic transvaginal ultrasound. On the second day, the hematology-oncology team initiated supportive care with two units of packed red blood cells and intravenous iron sucrose elevating her hemoglobin to 9 g/dL (Figure 2). A pelvic ultrasound revealed a fibroid within the uterine body measuring 2.3 x 1.7 x 1.9 cm. The OBGYN team discussed menorrhagia management options, including ablation and hysterectomy.

On the third day, she developed new symptoms of intermittent, crushing chest pain rated 10/10, tightness, and lower extremity pain. Diagnostic tests, including EKG, troponins, and D-dimer, were performed. Although her EKG showed normal sinus rhythm, troponins were negative, a chest X-ray showed no acute cardiopulmonary disease, and D-dimer was elevated at 628 ng/mL, concerning for a pulmonary embolism. A follow-up computed tomography (CT) angiography and cardiology consultation ruled out pulmonary embolism and acute coronary syndrome. A transthoracic echocardiogram revealed a preserved ejection fraction of 55-60% with no structural or functional abnormalities, and a leg ultrasound ruled out deep vein thrombosis. Ultimately, the HEME-ONC suggested acute chest syndrome was the culprit, and the patient was treated as a typical sickle cell disease with VOC. She received hydromorphone and methocarbamol for pain management.

By the fourth day, her hemoglobin dropped to 8.5 g/dL, prompting another prophylactic transfusion of packed red blood cells, after which her chest pain subsided. Her hemoglobin level rose to 9.6 g/dL post-transfusion. The plan was to continue intravenous iron sucrose and transition to oral iron in an outpatient setting and follow up for long-term management. The OBGYN team required a follow-up for the decision regarding the hysterectomy, and she was discharged on day 5 with this plan in place.

## Discussion

This report fundamentally reexamines the clinical understanding of Sickle-cell trait (SCT). Contrary to the historical view of SCT as a benign carrier state for Sickle Cell Disease (SCD), recent clinical cases and studies, including those presented here, suggest that SCT may have more serious implications under certain conditions. Specifically, in situations like hypoxic states or severe anemia, individuals with SCT may exhibit complications typically seen in SCD. While VOCs often manifest in SCT as splenic sequestration and infarction, our two patients presented with severe bilateral lower extremity pain which may be related to bone sequestration. This reevaluation is crucial, given the increased risk of venous thromboembolism, pulmonary embolism, kidney disease, exertional rhabdomyolysis, and high-altitude-related complications observed in SCT carriers. The mortality associated with exertional rhabdomyolysis in undiagnosed SCT athletes and military personnel has prompted mandatory screening programs, highlighting the evolving understanding of SCT’s potential health impacts. This changing perspective urges the importance of considering treatment regimens, typically reserved for SCD and vaso-occlusive crisis (VOC), for SCT carriers in specific clinical scenarios. Despite the general asymptomatic presentation of SCT carriers, the cases presented here underscore the potential for atypical and clinically significant events. Both of the presented cases describe females with a past medical history of SCT and uterine fibroids with heavy uterine bleeding. This poses a risk factor of heavy uterine bleeding exacerbating anemia which predisposes the patients to possible VOC. These events necessitate a comprehensive and discerning approach to SCT management, recognizing the infrequent but possible occurrence of vaso-occlusive phenomena.

## Conclusions

In conclusion, these cases contribute to the growing body of evidence suggesting that SCT, while generally less severe than SCD, is not without potential clinical complications. This emphasizes the importance of maintaining a high index of suspicion for bone sequestering VOCs in SCT carriers, especially in the presence of exacerbating factors like blood loss anemia.
